# THZ531 Induces a State of BRCAness in Multiple Myeloma Cells: Synthetic Lethality with Combination Treatment of THZ 531 with DNA Repair Inhibitors

**DOI:** 10.3390/ijms23031207

**Published:** 2022-01-21

**Authors:** Pavithra Shyamsunder, Shree Pooja Sridharan, Vikas Madan, Pushkar Dakle, Cao Zeya, Deepika Kanojia, Wee-Joo Chng, S. Tiong Ong, H. Phillip Koeffler

**Affiliations:** 1 Cancer Science Institute of Singapore, National University of Singapore, Singapore 117599, Singapore; shreepooja.ges@gmail.com (S.P.S.); vikasmadan@aol.com (V.M.); pushkar.dakle@gmail.com (P.D.); e0238008@u.nus.edu (C.Z.); dkanojia23@outlook.com (D.K.); mdccwj@nus.edu.sg (W.-J.C.); h.koeffler@cshs.org (H.P.K.); 2 Cancer & Stem Cell Biology Programme, Duke-NUS Medical School, Singapore 169857, Singapore; sintiong.ong@duke-nus.edu.sg; 3 Department of Hematology-Oncology, National University Cancer Institute of Singapore (NCIS), National University Hospital, Singapore 119074, Singapore; 4 Department of Haematology, Singapore General Hospital, Singapore 169608, Singapore; 5 Department of Medical Oncology, National Cancer Centre, Singapore 169610, Singapore; 6 Department of Medicine, Duke University Medical Center, Durham, NC 27710, USA; 7 Cedars-Sinai Medical Center, Division of Hematology/Oncology, UCLA School of Medicine, Los Angeles, CA 90095, USA

**Keywords:** DNA repair, BRCAness, THZ531, Olaparib

## Abstract

Multiple myeloma (MM) is a hematological disease marked by abnormal growth of B cells in bone marrow. Inherent chromosomal instability and DNA damage are major hallmarks of MM, which implicates an aberrant DNA repair mechanism. Studies have implicated a role for CDK12 in the control of expression of DNA damage response genes. In this study, we examined the effect of a small molecule inhibitor of CDK12–THZ531 on MM cells. Treatment of MM cells with THZ531 led to heightened cell death accompanied by an extensive effect on gene expression changes. In particular, we observed downregulation of genes involved in DNA repair pathways. With this insight, we extended our study to identify synthetic lethal mechanisms that could be exploited for the treatment of MM cells. Combination of THZ531 with either DNA-PK inhibitor (KU-0060648) or PARP inhibitor (Olaparib) led to synergistic cell death. In addition, combination treatment of THZ531 with Olaparib significantly reduced tumor burden in animal models. Our findings suggest that using a CDK12 inhibitor in combination with other DNA repair inhibitors may establish an effective therapeutic regimen to benefit myeloma patients.

## 1. Introduction

Multiple myeloma (MM) is a clonal B-cell malignancy that classically displays proliferation of plasma cells in the bone marrow accompanied by extensive chromosomal instability in those cells. Although the molecular basis of genomic stability is not fully understood, recently, it has been reported that the DNA damage response (DDR) may influence genomic changes in MM [1]. Defective DNA repair function provides an alternative explanation for aneuploidy and chromosomal rearrangements evidenced in MM cells [2]. These events further contribute to drug resistance in MM cells and thus defective DNA repair mechanisms have been implicated in the pathogenesis of MM [3,4,5]. DNA damaging chemotherapy has been the longstanding treatment strategy for cancer; however, it is accompanied by toxic side-effects and development of drug resistance over time. A new in line approach, synthetic lethality (SL), holds great promise in cancer therapeutics. SL provides avenues for drugging targets that are classically undruggable. The definition of SL has since been expanded to encompass pharmacologic inhibition of one gene product with inactivation of the other in cancer cells [6,7,8]. Seminal studies using SL approaches were used to target specific abnormalities in the DNA damage response (DDR) pathway in cancer cells for sensitization when administered in combination with DNA damaging agents [9,10]. The most prominent study is that of PARP inhibition as a means of triggering apoptosis in BRCA1 and BRCA2 defective tumors, which has significantly altered the treatment of breast and ovarian carcinomas [11,12]. Although new classes of drugs such as proteasome inhibitors and immunomodulatory drugs (bortezomib, thalidomide, and lenalidomide) have emerged in the past decade as great treatment modalities for MM [13,14], MM still remains largely a fatal disease with incurable proliferation of malignant plasma cells and thus identification of novel therapeutic agents is imminent. In the recent past, cyclin-dependent kinases (CDKs) have been cited as therapeutic agents for MM. [15,16,17]. Studies have shown that CDKs have been implicated to play a role in regulating homologous recombination (HR). CDK1 and 2 are known to phosphorylate BRCA1. These phosphorylation events are important for the formation of BRCA1 and RAD51 foci during DNA damage and HR [18,19]. In addition to CDK 1 and 2, CDK5 is overexpressed in MM cells and phosphorylates and activates ATM during S and G2-M checkpoint following DNA damage [20,21]. Given the prominent role of these CDKs in DNA damage response, cells depleted of CDK1 or 5 are sensitive to PARP inhibitors [22], and overexpression of CDK5 mediates chemo resistance [23]. Given the role CDKs play in DNA repair by HR, CDK inhibitors such as flavopiridol, AG024322, and AZD5438 have been shown to impair HR function, and sensitize cancer cells to DNA-damaging agents, including PARP inhibitors [24,25,26]. Extensive genomic instability and elevated homologous recombination activity in MM opens the potential for a greater therapeutic window by inhibiting the genes involved in the DDR machinery. Dinacilib, a small molecule inhibitor of CDKs 1, 2, 5, and 9 has been indicated to disrupt HR repair in MM [27]. In this study, we assessed the role of THZ531, a small molecule inhibitor of CDK12 and 13 in MM. We identified profound changes in the transcript levels of DDR genes post THZ531 treatment and followed up with synthetic lethal approaches of combining THZ531 along with potent DNA repair pathway inhibitors in clinical use. We provide evidence that THZ531 could disrupt DNA repair pathway in MM cells, leading to contextual synthetic lethality if combined with DNA repair inhibitors such as PARP inhibitors.

## 2. Results

### 2.1. Multiple Myeloma Cells Are Highly Sensitive to THZ531 Inhibition

**Figure 1:**
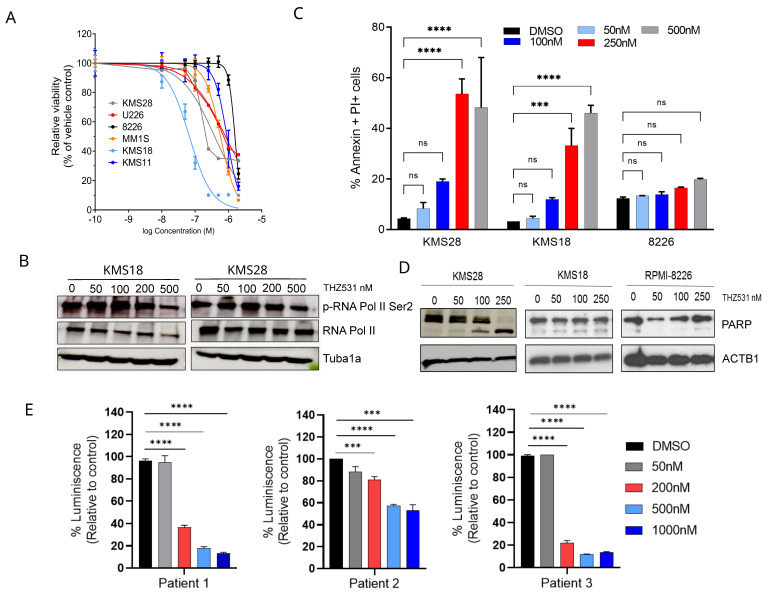
Multiple myeloma cells are highly sensitive to THZ531. (**A**) A diverse panel of multiple myeloma cells lines were treated with either DMSO (control) or increasing concentrations of THZ531. Cell viability was assessed 72 h following treatment. (**B**) Western blot depicting the effect of THZ531 on p-RNA Pol II Ser 2 in2 myeloma cell lines. Alpha tubulin was the loading control. (**C**) Effect of increasing concentration of THZ 531 on the percentage of Annexin V+ cells measured via flow cytometry at 24 h. (**D**) Western blot depicting the effect of THZ 531 on PARP cleavage at 24 h. Beta-actin was used as the loading control. (**E**) Effect of THZ531 on 3 multiple myeloma patient samples (CD138+). Samples were treated with THZ531 for 48 h, and viability was measured using Cell titre glo assay. Data for (**C**,**E**) are presented as the mean ± SD of the mean. Statistical analysis was carried out using GraphPad Prism software, version 7 (GraphPad, La Jolla, CA, USA). Statistical significance was determined using an unpaired Student’s *t*-test or ANOVA followed by Tukey’s post hoc test. ns—not significant; *p* < 0.05 considered significant. *** *p* < 0.0002, **** *p* < 0.0001.

THZ531, a covalent and potent inhibitor of CDK12 and 13, has been shown to be effective in Ewing’s sarcoma. Analysis of a biomarker screen carried out by Iniguez et al., revealed high sensitivity of MM cells to CDK inhibitor treatment [28]. On screening a panel of MM cells with THZ531, we noticed decreased cell viability with most MM cell lines tested (Figure 1A). To measure the activity of THZ531, we examined levels of its target pSer 2 RNA Pol II in MM cell lines with increasing concentrations of THZ531. THZ531 modestly suppressed RNA Pol II phosphorylation at Ser 2 in a dose-dependent manner (Figure 1B, Supplementary Figure S1A, see Supplementary Materials). Apoptotic assays after THZ531 treatment showed an increase in Annexin + PI+ cells in KMS18 and KMS 28 (100–200 nM—using sensitive MM cell lines to THZ531) in comparison to RPMI-8226 (non-responsive cell line) (Figure 1C). Findings of the apoptotic assays were also validated through Western blotting for PARP cleavage, wherein cleaved PARP product occurred with increasing concentrations of THZ531 (Figure 1D). In order to prove that the anti-growth effect seen after THZ531 treatment was due to CDK12 inhibition, shRNA knock-down of CDK12 was done in MM cells lines (Supplementary Figure S1B). Genetic suppression of CDK12 dramatically decreased cell viability of MM cells (Supplementary Figure S1C). Extending this to primary MM samples (purified for CD138+ cells) treated with THZ531 decreased growth of MM cells (Figure 1E). Collectively, these results demonstrate the dependency of MM cells on CDK12 and the effectiveness of THZ531 treatment for MM.

### 2.2. THZ531 Treatment Represses Genes Involved in DNA Repair and Induces a State of BRCAness

**Figure 2:**
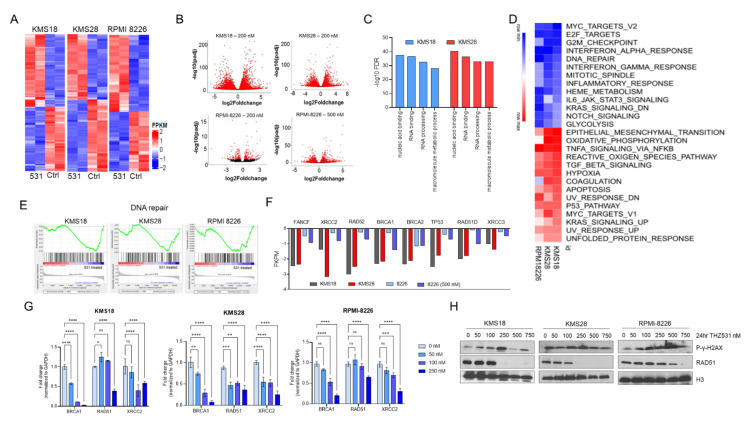
THZ531 treatment downregulates genes involved in DNA repair pathways and induces DNA damage in MM cells (**A**) KMS18, KMS28, and RPMI-8226 were treated with 200 nM THZ531 for 6 h in duplicates. Heatmap displays the log2 fold change in gene expression for THZ531 versus DMSO treatment. (**B**) Volcano plots depicts the effect of 200 nM THZ531 on gene expression. In addition, 500 nM THZ531 on gene expression changes of RPMI-8226 cells is also depicted. (**C**) Gene ontology analysis of genes dysregulated with 200 nM THZ531 treatment of KMS18 and KMS28 cells. (**D**) Heat map of GSEA pathways enriched in MM cells post 6 h of THZ531 treatment. (**E**) GSEA plots for DNA repair pathway gene sets among the downregulated genes 6 h after 200 nM THZ531 treatment. (**F**) FPKM values of genes involved in DNA repair pathways that were downregulated following THZ531 treatment. (**G**) Quantitative real time PCR validation of a set of DNA repair pathway genes treated with increasing doses of THZ 531 (0, 50, 100, 250 nM). Data presented as the mean ± SD of the mean. Statistical analysis was carried out using GraphPad Prism software (GraphPad, La Jolla, CA). Statistical significance was determined ANOVA followed by a Tukey’s posthoc test. ns—not significant, ns- not significant, * *p* < 0.05 ** *p* < 0.001, *** *p* < 0.0002, **** *p* < 0.0001. (**H**) Immunoblotting for phosphor-gamma H2AX and RAD51 in KMS18, KMS28, and RPMI-8226 treated with increasing doses of THZ531. Histone H3 and GAPDH were used as loading controls.

Treatment of cells with CDK inhibitors causes a massive change in gene expression profiles [28]. We investigated the effect of THZ531 on global gene expression using RNA sequencing. Using two highly sensitive MM cell lines (KMS 18 and KMS 28) and one non-sensitive cell line (RPMI-8226), samples were treated in duplicate with either diluent (DMSO) or THZ531 (200 and 500 nM for 6 h). THZ531 downregulated steady-state mRNA levels in a concentration-dependent manner (Figure 2A) with profound gene expression changes at 200 and 500 nM for KMS18 and KMS28 (4000 genes downregulated), while RPMI 8226 had just a modest effect at 200 nm (1170 downregulated genes); and at a higher dose of 500 nM, the effect was more pronounced (2075 genes downregulated) (Figure 2B). Gene expression changes between KMS18 and KMS28 were strongly correlated (Supplementary Figure S2). In order to understand the pathways that are differentially expressed between THZ531 sensitive and resistant cell lines, we carried out functional enrichment of genes that were differentially expressed in KMS18 and KMS28, but not in RPMI-8226. Notably we observed enrichment of pathways implicated in NFkB pathway and p53 signaling. GO analysis also revealed enrichment of genes involved in negative regulation of G1/S transition in THZ531-sensitive cells. The results of this analysis have been reported in Supplementary Table S1. Gene ontology analysis of highly downregulated genes (log fold change < 1) revealed over- representation of genes involved in transcription and replication as well as genes involved in DNA repair pathway, especially those involved in HR pathway (Figure 2C). GSEA analysis of down-regulated genes identified enrichment of MYC, E2F targets and those involved in cell cycle and DNA damage response pathways (Figure 2D). Given the inherent genomic instability of MM cells, we chose to study the implication of DNA repair gene downregulation further. All three cell lines used in the study had enrichment of DNA repair pathway (Figure 2F). Leading edge of the enriched GSEA pathway revealed involvement of genes involved in HR pathway. Figure 2E depicts the RNA seq fpkm values for genes involved in HR pathway. We validated the RNA seq data by treating cells with three doses of THZ531 and carried out Quantitative PCR analysis for these identified HR pathway genes (Figure 2G). In order to verify whether treatment with THZ531 induced an inherent DNA damage response, phosphorylated γ-H2AX was examined in MM cell lines treated with increasing doses of THZ531. A concentration dependent accumulation of phosphorylated γ-H2AX occurred in all the cell lines treated with THZ531 (Figure 2H). In addition, we also noted significant downregulation of RAD51 protein levels after THZ531 treatment (Figure 2H). RAD51 and its paralogs are known to play a central role in HR repair [29]. In addition, CDK inhibitors are known to cause p53-mediated downregulation of RAD51 and thus result in HR impairment and sensitivity DNA-damaging agents [30,31]. Together, these findings suggest that treatment of MM cells with THZ531 affects global gene expression, affecting particularly the HR DNA damage pathway inducing a state of BRCAness in MM cells.

### 2.3. Specific Targeting of NHEJ and BER Pathway Is Effective in MM Cells

**Figure 3:**
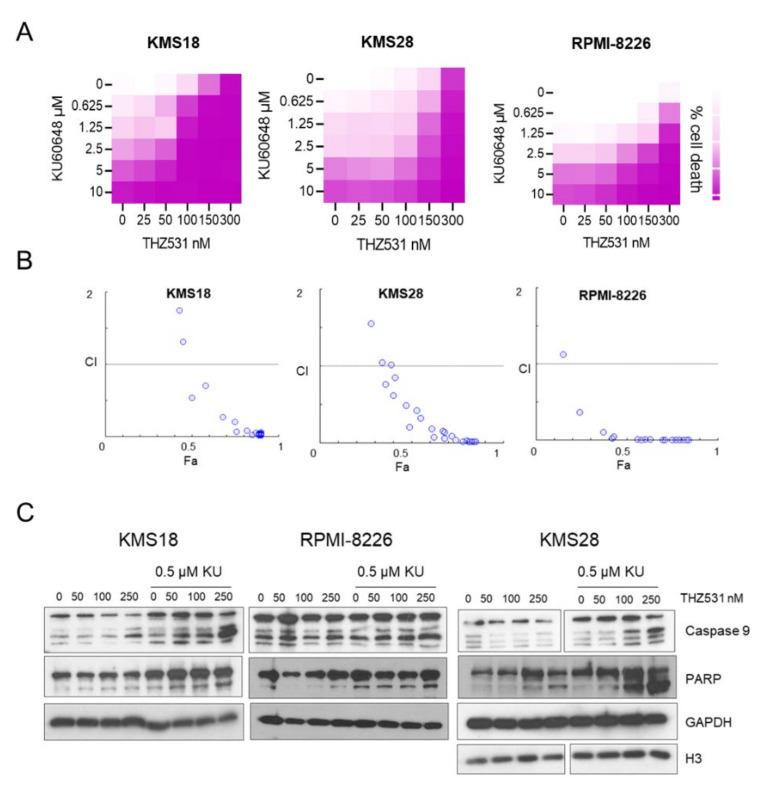
Synergistic effect of DNA-PK inhibitor KU-0060648 with THZ531: (**A**) MTT assay to predict synergistic growth inhibition of KMS18, KMS28, and RPMI-8226 MM cells in the presence of THZ531 and DNA-PK inhibitor KU-0060648. (**B**) Synergy and CI prediction of combination treatment. The combination index (CI) defines interaction between THZ531 and KU-0060648 plotted as a fraction of cell viability. CI < 1, CI = 1, and CI > 1 represent synergism, additive effects, and antagonism, respectively, of the two compounds. (**C**) Immunoblotting for cleaved Caspase-9 and cleaved PARP in KMS18, KMS28, and RPMI-8226 treated with increasing doses of THZ531 (0, 50, 100, 250 nM) alone or in combination with 0.5 µM KU-0060648. GAPDH and H3 were used as loading control. The THZ531 PARP blot shown in Figure 3C (KMS18 and RPMI-8226) was derived from the same experiment as that in Figure 1D, reused here to demonstrate the KU-combination condition.

As THZ531 treatment downregulated HR pathway genes and given the sensitivity of MM cells to DNA, alkylating agents’ combination treatment of THZ531 with DNA repair pathway inhibitors was tested. Cells with a defective HR repair pathway try to correct DNA damage by utilizing other modes of DNA repair pathways such as non-homologous end joining (NHEJ) and base excision repair (BER). DNA-PK inhibitors have emerged as successful agents to target NHEJ mechanism given their efficacy in MM cells [32,33]. We tested combination of DNA-PKi (KU-0060648) and THZ531 on MM cells. Cells were treated with increasing concentration of both drugs as single agents and in combination for 72 h. Post 72 h, the plates were measured and viability was computed (Figure 3A). First column of Figure 3A served as increasing dose of KU-0060648 as a single agent and last row of Figure 3A served as increasing dose of THZ531 as a single agent. Every other cell in the matrix received respective combinations of KU-0060648 and THZ531. Single agent treatment with KU-0060648 had no effect on cell viability. In contrast, combination treatment of KU-0060648 with THZ531 led to cell death (Figure 3A). We further interrogated these viability readings to understand whether combination treatment was synergistic. Compusyn software was used for synergy and combination index (CI) predictions. CI < 1, CI = 1, and CI > 1 represent synergism, additive, and antagonism, respectively, of the two compounds. Interestingly, we observed that most of the drug combinations were synergistic (Figure 3B). The actual concentrations of drugs that led to synergistic cell killing have been listed in Supplementary Table S2. Of specific note, RPMI-8226 cells, which are insensitive to THZ531 treatment, responded synergistically with combination treatment. We further validated KU-0060648 and THZ531 synergy by carrying out Western blotting for PARP and Caspase-9. We observed that combination treatment also increased cleaved PARP and Caspase-9 products (Figure 3C).

**Figure 4:**
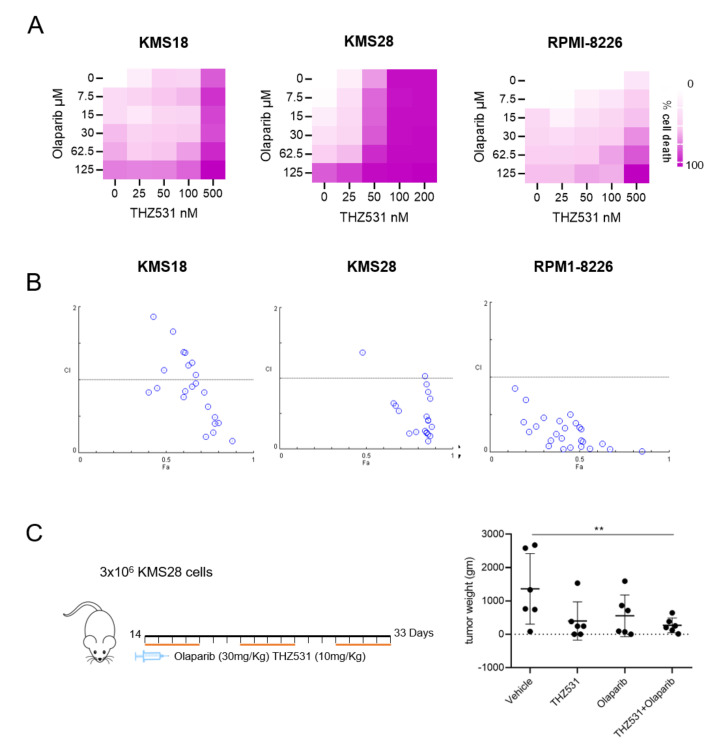
THZ531 and Olaparib are synergistic in multiple myeloma cells. (**A**) MTT assay of KMS18, KMS28, and RPMI-8226 multiple myeloma cells in the presence of THZ531 and PARP inhibitor Olaparib. (**B**) Combination index (CI) prediction of Olaprib and THZ531 treatment. CI defines interaction between the two compounds. CI < 1, CI = 1, and CI > 1 represent synergism, additive, and antagonism. (**B**) NSG mice were injected subcutaneously with 3 × 10^6^ KMS 28 cells, and mice were randomized into 4 groups (*n* =6 mice per group). When tumors became palpable, groups were treated with vehicle (DMSO), THZ531 (10 mg/kg i.p. five times a week), Olaparib (30 mg/kg oral gavage, five times a week), or the combined treatments of THZ531 and Olaparib. (**C**) Volume measurement of harvested tumors, 3 weeks post treatment. ** *p* < 0.001.

BER pathway is another mode of DNA repair that cells use to protect themselves against DNA breaks. PARP complex is an essential member of the BER complex. Treatment of MM cells with various PARP inhibitors as single agents induces DNA DSBs that are effectively repaired via HR; thus, they do not affect cell viability [34]. We noted that THZ531 impaired HR repair by downregulating vital HR repair genes, therefore, we rationalized that combination of THZ531 with a PARP inhibitor might synergistically kill multiple myeloma cells. Similar to the methodology above, we treated MM cells with increasing concentration of PARPi (Olaparib) and THZ531, as single agents and in combination for 72 h. Cell viability of MM cells was assessed by treatment with either PARP inhibitor (Olaparib) or THZ531, versus their combination. Treatment with Olaparib (2–20 μmol/L) alone had no significant effect on cell viability against any of the MM cell lines; however, in combination treated wells we observed enhanced cell death (Figure 4A). Further, we computed CI values for Olaparib and THZ531 combination. All three cell lines tested revealed synergy with combination treatment (Figure 4B). The individual values of drug dosage and CI index have been provided in Supplementary Table S2. Next, the combination was tested in vivo in immune-deficient NSG mice. The effects of Olaparib (30 mg/kg by oral gavage once daily, five days per week) and/or THZ 531 (10 mg/kg i.p., once daily, five days per week) was tested against KMS28 subcutaneous tumors (Figure 4B). On the third week of treatment, tumors were weighed (Figure 4C) and tumor images have been provided in Supplementary Figure S3. Significant synergy was observed in combination treated group. Slight weight loss occurred in mice treated with THZ531, but all mice were healthy and active, not showing signs of toxicities. Taken together, our studies indicate that targeting of multiple DNA repair pathways can have synthetic lethality for MM cells and this should be examined in clinical trials of the disease.

## 3. Discussion

Intrinsic DNA damage and chromosomal instability are hallmarks of MM resulting in perpetual accumulation of genetic alterations that lead to progression of the disease. Although the mechanism for this genomic instability is not clearly understood, recent studies have proposed dysregulated DNA damage repair (DDR) pathways to be the driving cause of these abnormalities. HR activity is reported to be elevated in MM cells, thus leading to an increased rate of mutational burden over time and eventual development of drug resistance [3]. Hence, treatment of MM remains challenging, and despite numerous advances, an unmet need for alternative treatment strategies are required. CDK12 regulates the expression of HRR genes, thereby maintaining genomic stability. Cells expressing catalytically inactive forms of CDK12 display lowered ability to effectively carry out HR [35]. Murine blastocysts deficient of CDK12 display decreased expression of HR repair genes and increased levels of DNA damage [36]. CDK12 loss-of-function mutations are detected in malignancies with highly unstable genome, such as high-graded serous ovarian carcinomas and metastatic-castration resistant prostate cancers [37,38,39]. Wildtype CDK12 has vulnerability in EWS/FLI-positive Ewing sarcoma cells treated with THZ531, leading to downregulation of HRR genes [28]. Given the sensitivity of MM cells to CDK12 depletion, we postulated that this sensitivity to CDK12 inhibition could be an outcome of heightened genomic instability of MM. CDK12 exerts its function by mediating the phosphorylation of Ser2 on the C-terminal domain (CTD) of RNA polymerase II, which is necessary for transcription of HRR genes [40]. Consistent with this model, we demonstrated that THZ531 reduced in a concentration-dependent manner pSer2. In addition, silencing CDK12 led to a dramatic reduction in cell viability of MM cells, confirming that CDK12 is required for cell survival. Moreover, gene expression data identified that THZ531 preferentially downregulated DDR genes, especially those involved in HR pathway (BRCA1, BRCA2, FANCF, RAD51, and its paralogs RAD51C, RAD51D, and XRCC2).

THZ531 altered the phosphorylation of γ-H2AX and decreased both mRNA and protein levels of RAD51, a core member essential for HR repair [41]. Inhibition of HR pathway causes cells to salvage DNA DSBs by utilizing the NHEJ pathway involving the DNA-PK proteins [42]. Apart from DNA damage response, DNA-PK activity is necessary for multiple cellular functions, including regulation of transcription, progression of the cell cycle, and maintenance of telomeres [43,44,45]. They have emerged as successful agents to target NHEJ mechanism in several cancer types including MM [32,33]. We observed that THZ531 produced synergistic cell death with the DNA-PK inhibitor KU-0060648.

Previous studies demonstrated the inability of PARP inhibitors alone to decrease significantly the survival of MM cell lines because HR function effectively repaired the induced DSBs [34]. However, specific depletion of CDKs caused hypersensitivity of tumor cells to PARP inhibition through HR deficiency and consequent increase in DNA DSBs [18,19,46]. Dinaciclib (CDK 1, 2, 5, and 9 inhibitor) leads to the impairment of HR pathway and produces synergistic cell killing when combined with PARP inhibitor, ABT-888 [27]. Similar effects were observed in BRCA-active breast cancer; Dinaciclib was more potent against CDK12 than the other target CDKs, leading to sensitization of tumor cells to PARP inhibition by Veliparib [47]. Furthermore, CDK12 specific inhibition in EWS/FLI-positive Ewing sarcoma cells using THZ531 prominently synergized with ABT-888 [28]. Although we found that THZ531 significantly decreases MM cell viability, a synthetic lethal effect ensued when combined with Olaparib, resulting in a significantly greater loss of viability relative to single agent administration. This synthetic lethal effect was evidenced in MM xenografts in NSG mice treated with THZ531 (10 mg/kg) and/or Olaparib (30 mg/kg). After three weeks of treatment, the tumor weight was found to be significantly reduced in the combination compared with each individual agents. Our study showed that (THZ531) decreased myeloma cell growth especially when combined with other DNA damaging agents, probably by disrupting HR function of MM cells. We therefore conclude that THZ531 impaired HR-mediated repair of DNA damage causing sensitivity of MM cells to DNA repair inhibitors such as either PARP inhibitor or DNA-PK inhibitor. In addition to Ewing’s sarcoma, THZ531 has also shown to have synergistic effect with sorafenib in the treatment of hepatocellular carcinoma [48]. Although the toxicity of THZ531 on normal cells has not been documented yet, all these studies, including ours, emphasize on the importance of CDK12 inhibitors and their potential in cancer therapeutics. In summary, our study provides a rationale for this novel combination treatment for MM and the framework for further investigation of its safety and efficacy in MM patients.

## 4. Materials and Methods

### 4.1. Cell Culture and Lentiviral Transduction

Human MM cell lines: KMS11, KMS28, KMS18, KMS12, MM1S, MM1R, H929, 8226, 8226 LR5, and 8226 P100V were kind gifts from Dr. W.J. Chng (Cancer Science Institute of Singapore, Singapore). All cell lines were maintained in RPMI 1640 supplemented with 10% fetal bovine serum (Thermo Scientific, Waltham, MA, USA) and kept in a humidified incubator at 37 °C with 5% CO_2_. To obtain MM cells with stable knock-down of CDK12, cells were transduced with lentiviral particles with shRNA against CDK12 at 1000 g for 90 min in the presence of 8 µg/mL polybrene (Sigma-Aldrich, St. Louis, MO, USA). Stable cells with CDK12 knock-down were selected in 1 µg Puromycin medium.

### 4.2. MTT Assay

For viability assays, cells were seeded in 96-well plates either in the presence or absence of different concentrations of inhibitors for 72 h. After 72 h culture, 10 μL of MTT (2-(4,5-dimethylthiazol-2-yl)-2,5-diphenyltetrazolium bromide) (Sigma-Aldrich, St. Louis, MO, USA) was added to the wells and cultured at 37 °C for an additional 4 h followed by addition of 100 μL stop solution. Plates were measured using a Tecan plate reader at 570 nM absorbance. Calculation of IC50 was based on sigmoidal dose-response curve fitting, using Graphpad prism (La Jolla, CA, USA). Combination index (CI) was calculated using Calcusyn software. Values of CI indicate synergistic (<1), additive (=1), or antagonistic (>1) effects. 

### 4.3. Cell Titre Glo Assay

Three primary CD138+ purified MM patient samples were plated in 3 independent wells of a 96-well plate with different concentrations of either THZ531 or DMSO as a vehicle. At 48 h post initiation of treatment, cell viability was determined using the CellTiter-Glo^®^ luminescent cell viability kit (Promega, Madison, WI, USA) according to the manufacturer’s instructions. The luminescence was measured using GloMax^®^-Multi Detection System (Madison, WI, USA). Means with standard deviations are shown.

### 4.4. RNA Isolation, Reverse Transcription, and Quantitative RT-PCR

RNA was isolated using RNeasy Mini Kit (Qiagen, Hilden, Germany), according to manufacturer’s instructions. cDNA was prepared using EVO Script reverse transcriptase (Roche, Basel, Switzerland). Primer sequences used for quantitative RT-PCR are listed in Supplementary Table S3.

### 4.5. RNA Sequencing and Gene Expression Analysis

For RNA sequencing, cDNA libraries were prepared from poly-A selected RNA using Truseq RNA sample kit (Illumina). Libraries were sequenced on HiSeq 4000 and 100 bp paired-end reads were aligned to human reference transcriptome using Kallisto (version 0.43.0) [49]. Kallisto results were imported into DESeq2 using tximport Bioconductor package [50]. Differential gene expression analysis was performed using DESeq2 with lfcThreshold argument set to 0.1. All other test-statistics and plotting were performed using R 3.4.0. Expression values were calculated in terms of FPKM for every gene with DESeq2::fpkm function. Gene ontology (GO) was performed on differentially expressed genes using goseq Bioconductor package (version 1.20.0). Resulting p-values were adjusted for false discovery rate (FDR). Gene set enrichment analysis (GSEA) was performed on all active genes (mean FPKM  >  0.5)to identify enriched gene sets among MSigDB C2 gene sets [51].

### 4.6. Immunoblotting

MM cells were lysed in 2X gel loading dye, and proteins were resolved on 10–12% SDS-PAGE gel. Proteins were transferred to PVDF membranes and probed with primary antibodies overnight. Membranes were incubated with appropriate HRP-conjugated secondary antibodies for an hour and developed using SuperSignal West Femto Maximum Sensitivity Substrate (Thermo Fisher Scientific, Waltham, MA, USA). Antibodies used are listed in Supplementary Table S1.

### 4.7. Apoptotic Assay

Cells were seeded in 6 well plates either in the presence or absence of different concentrations of inhibitors. A total of 48 h after seeding, staining was performed using Apoptosis Detection Kit II (BD Biosciences, New Jersey, USA). Cells were harvested and washed twice with phosphate-buffered saline (Thermo Fisher Scientific, Waltham, MA, USA), suspended in 1X binding buffer with 5 μL of Alexa Fluor 488 conjugated Annexin V and 5 μL of PI for 15 min in the dark at room temperature. A total of 10,000 events were captured per sample. Flow cytometric analysis was performed on a FACS LSR II flow cytometer. Cells positive for Annexin and PI were defined as apoptotic cells. Data were analyzed using FACSDiva software (BD Biosciences, NJ, USA).

### 4.8. Animal Studies

All animal studies were in accordance with protocols approved by Institutional Animal Care and Use Committee (IACUC) at National University of Singapore. NSG mice were purchased from InVivos, Singapore. The mice were kept at Animal Research Facilities at National University of Singapore in a sterile condition at 20–26 °C temperature, 50% humidity, and in a light-controlled environment (12 h light, 12 h dark). Mice were provided food and water ad libitum. They were monitored daily by trained comparative medicine staff for their health and well-being. For the xenograft experiments, MM cell line KMS-28 (3 × 10^6^ cells) were injected subcutaneously into six-week-old NSG mice. Mice were randomly divided into four groups: (1) Oral treatment with Olaparib (30 mg/kg); (2) Intraperitoneal injection with THZ 531 (10 mg/kg); (3) Treatment with both Olaparib and THZ531; (4) Diluent control. The mice received the drug/diluent control five times per week for 3 weeks.

### 4.9. Patient Sample Analysis

All experiments involving patient samples was conducted according to the guidelines of the Declaration of Helsinki, and approved by the Institutional Review Board Genomic-Based Diagnosis, Classification and Targeted Treatment of Multiple Myeloma- DSRB Ref 2012/00058.

### 4.10. Statistical Analysis

Appropriate statistical analysis was carried using Graph Pad Prism (La Jolla, USA). Data represented as mean ± SD. *p*-values < 0.05 are considered statistically significant.

## Data Availability

Not applicable.

## Supplementary Materials

The following are available online at https://www.mdpi.com/article/10.3390/ijms23031207/s1.
