# Correction: Shyamsunder et al. THZ531 Induces a State of BRCAness in Multiple Myeloma Cells: Synthetic Lethality with Combination Treatment of THZ 531 with DNA Repair Inhibitors. *Int. J. Mol. Sci.* 2022, *23*, 1207

**DOI:** 10.3390/ijms27125312

**Published:** 2026-06-12

**Authors:** Pavithra Shyamsunder, Shree Pooja Sridharan, Vikas Madan, Pushkar Dakle, Cao Zeya, Deepika Kanojia, Wee-Joo Chng, S. Tiong Ong, H. Phillip Koeffler

**Affiliations:** 1 Cancer Science Institute of Singapore, National University of Singapore, Singapore 117599, Singapore; shreepooja.ges@gmail.com (S.P.S.); vikasmadan@aol.com (V.M.); pushkar.dakle@gmail.com (P.D.); e0238008@u.nus.edu (C.Z.); dkanojia23@outlook.com (D.K.); mdccwj@nus.edu.sg (W.-J.C.);; 2 Cancer & Stem Cell Biology Programme, Duke-NUS Medical School, Singapore 169857, Singapore; sintiong.ong@duke-nus.edu.sg; 3 Department of Hematology-Oncology, National University Cancer Institute of Singapore (NCIS), National University Hospital, Singapore 119074, Singapore; 4 Department of Haematology, Singapore General Hospital, Singapore 169608, Singapore; 5 Department of Medical Oncology, National Cancer Centre, Singapore 169610, Singapore; 6 Department of Medicine, Duke University Medical Center, Durham, NC 27710, USA; 7 Cedars-Sinai Medical Center, Division of Hematology/Oncology, UCLA School of Medicine, Los Angeles, CA 90095, USA

In the original publication [1], we used the same blot in two figures (Figures 1D and 3C), but the accompanying figure legend does not match. We wish to include a few lines in the existing legend. The correct legend appears below:

**Figure 3.** Synergistic effect of DNA-PK inhibitor KU-0060648 with THZ531: (**A**) MTT assay to predict synergistic growth inhibition of KMS18, KMS28, and RPMI-8226 MM cells in the presence of THZ531 and DNA-PK inhibitor KU-0060648. (**B**) Synergy and CI prediction of combination treatment. The combination index (CI) defines interaction between THZ531 and KU-0060648 plotted as a fraction of cell viability. CI < 1, CI = 1, and CI > 1 represent synergism, additive effects, and antagonism, respectively, of the two compounds. (**C**) Immunoblotting for cleaved Caspase-9 and cleaved PARP in KMS18, KMS28, and RPMI-8226 treated with increasing doses of THZ531 (0, 50, 100, 250 nM) alone or in combination with 0.5 µM KU-0060648. GAPDH and H3 were used as loading control. The THZ531 PARP blot shown in Figure 3C (KMS18 and RPMI-8226) was derived from the same experiment as that in Figure 1D, reused here to demonstrate the KU-combination condition.

There was also a mistake in the original publication [1]: a correction has been made to Figure 1B. After re-examining the original image files, we confirmed that an inadvertent mix-up occurred during figure assembly. Specifically, the RNA Pol II Ser2 and RNA Pol II panels for KMS18 and KMS28 were incorrectly interchanged. A revised Figure 1B has been generated directly from these raw blots. We sincerely apologize for this unintentional oversight. The correct Figure 1 appears below.

The authors state that the scientific conclusions are unaffected. These corrections were approved by the Academic Editor. The original publication has also been updated.

## Figures and Tables

**Figure 1:**
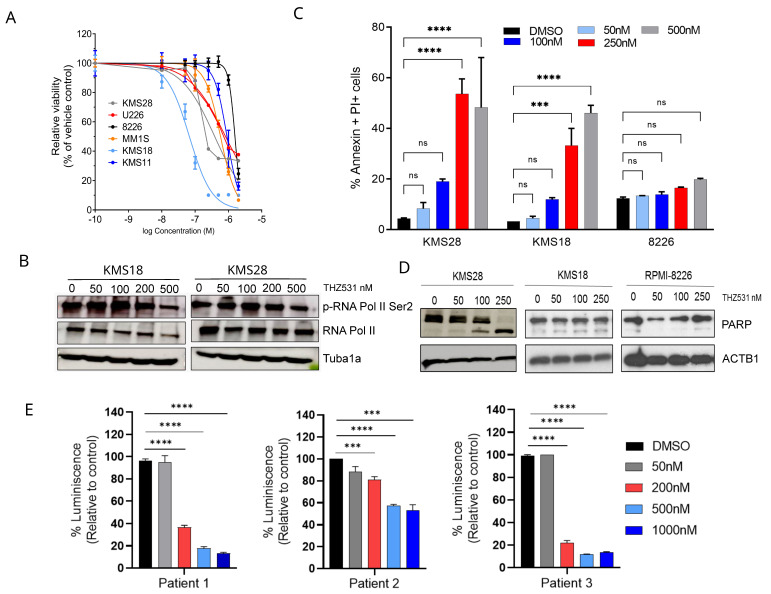
Multiple myeloma cells are highly sensitive to THZ531. (**A**) A diverse panel of multiple myeloma cells lines were treated with either DMSO (control) or increasing concentrations of THZ531. Cell viability was assessed 72 h following treatment. (**B**) Western blot depicting the effect of THZ531 on p-RNA Pol II Ser 2 in2 myeloma cell lines. Alpha tubulin was the loading control. (**C**) Effect of increasing concentration of THZ 531 on the percentage of Annexin V+ cells measured via flow cytometry at 24 h. (**D**) Western blot depicting the effect of THZ 531 on PARP cleavage at 24 h. Beta-actin was used as the loading control. (**E**) Effect of THZ531 on 3 multiple myeloma patient samples (CD138+). Samples were treated with THZ531 for 48 h, and viability was measured using Cell titre glo assay. Data for (**C**,**E**) are presented as the mean ± SD of the mean. Statistical analysis was carried out using GraphPad Prism software, version 7 (GraphPad, La Jolla, CA, USA). Statistical significance was determined using an unpaired Student’s *t*-test or ANOVA followed by Tukey’s post hoc test. ns—not significant; *p* < 0.05 considered significant. *** *p* < 0.0002, **** *p* < 0.0001.
