# Supplementary material for: THZ531 Induces a State of BRCAness in Multiple Myeloma Cells: Synthetic Lethality with Combination Treatment of THZ 531 with DNA Repair Inhibitors
Source: Int J Mol Sci. 2022 Jan 21;23(3):1207. doi: 10.3390/ijms23031207 (PMC8835885; doi:10.3390/ijms23031207)
Supplement: Supplementary file 1 [file ijms-23-01207-s001.zip › Supplementary data/Supplementary Figures.pdf]

Supplementary Figure S1

A

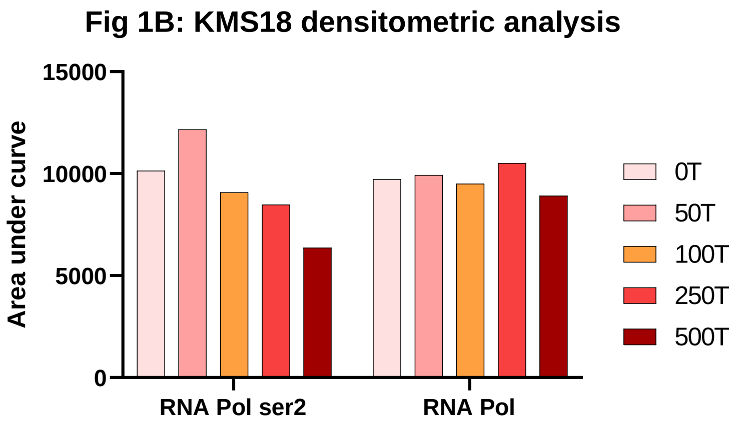

B

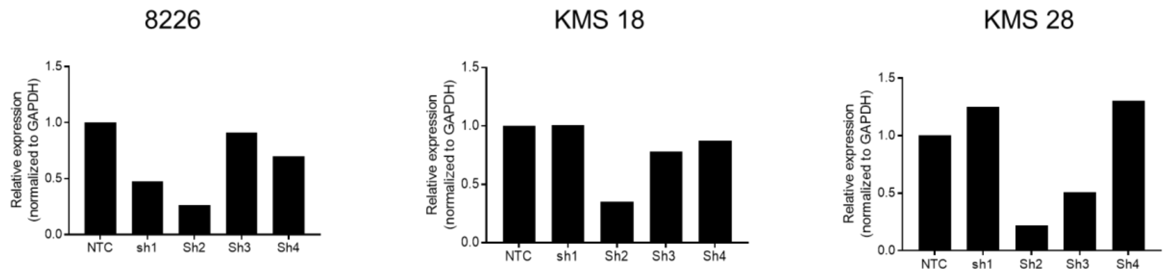

C

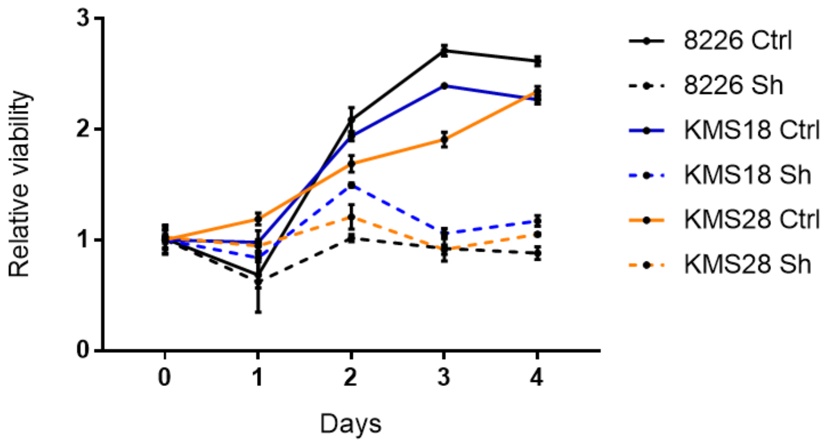

Supplementary Figure S1: (**A**) Densitometry analysis of western blot for RNA Pol and Ser2 of KMS18 cells treated with increasing concentration of THZ531 (**B**) Quantitative real time PCR of CDK12 expression in MM cells transduced with shRNA's against CDK12. (**C**) Viability of CDK12-Sh2 transduced MM cells in comparison to non-targeting control.

Supplementary Figure S2

A

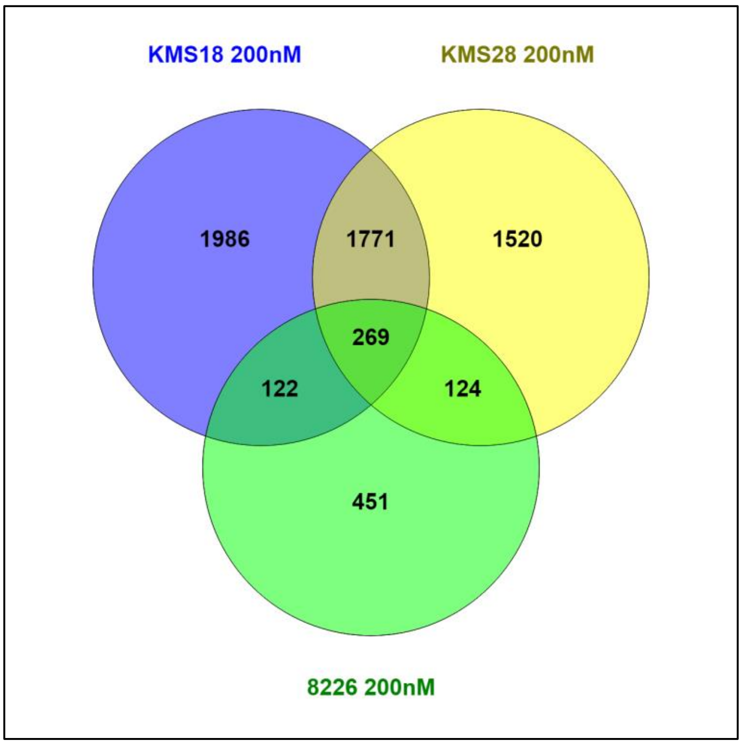

B

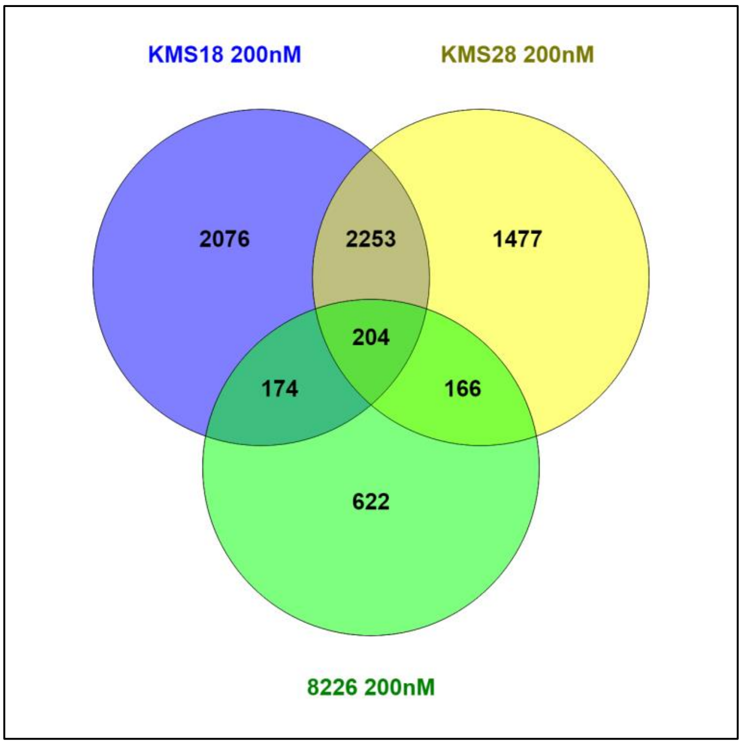

Supplementary Figure S2: (**A**) Overlap of upregulated genes (**B**) downregulated genes after THZ531 treatment.

Supplementary Figure S3

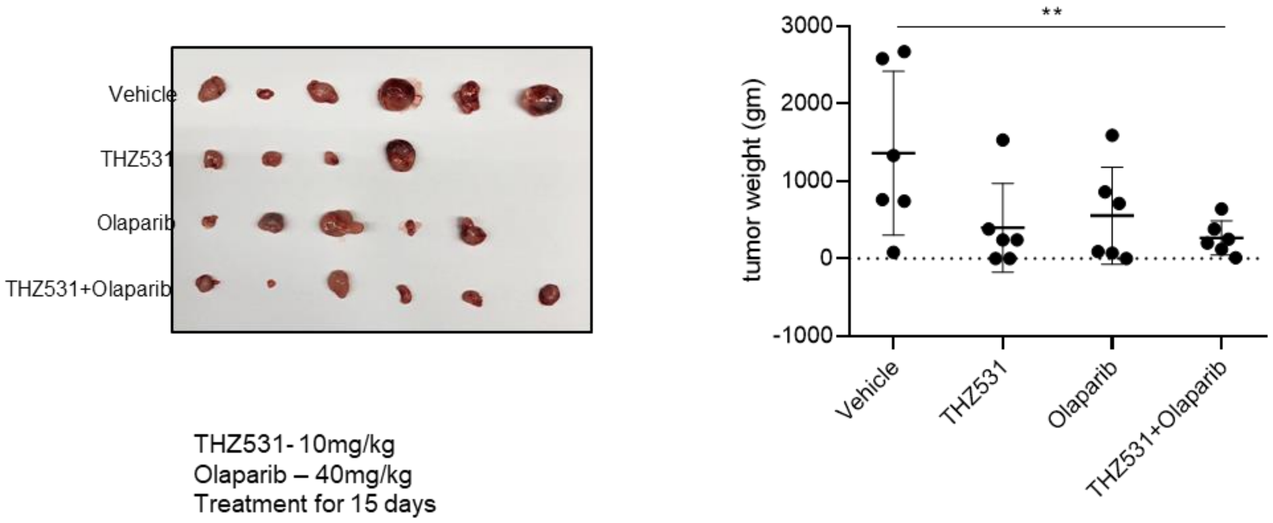

Supplementary Figure S3: Tumors harvested from mice treated with vehicle, THZ531, Olaparib and combination of THZ531 and Olaparib.
