# Supplementary material for: THZ531 Induces a State of BRCAness in Multiple Myeloma Cells: Synthetic Lethality with Combination Treatment of THZ 531 with DNA Repair Inhibitors
Source: Int J Mol Sci. 2022 Jan 21;23(3):1207. doi: 10.3390/ijms23031207 (PMC8835885; doi:10.3390/ijms23031207)
Supplement: Supplementary file 1 [file ijms-23-01207-s001.zip › Supplementary data/Supplementary Table 3.pdf]

Supplementary Table S3. List of primers and shRNAs used in this study.

RT-PCR Primers

BRCA1 FP	TTCACCCTCTGCTCTGGGTA
BRCA1 RP	TGGTCACACTTTGTGGAGACA
RAD51 FP	CAATGCAGATGCAGCTTGAA
RAD51 RP	CCTTGGCTTCACTAATTCCCT
XRCC2 FP	CAGTTGGTGAATGGCGTTGG
XRCC2 RP	TACCTTCAAGTCGGGCAAGG
GAPDH FP	CTCCTCCTGTTCGACAGTCAGC
GAPDH RP	CCATGGAATTTGCCATGGGTGG

shRNA target sequences

CDK12 Sh1	CCGGGCGTTCCCGGGACTTACTAAACTCGAGTTTAGTAAGTCCCGGGAACGCTTTTTG
CDK12 Sh2	CCGGTTGGACTTGCTCGGCTCTATACTCGAGTATAGAGCCGAGCAAGTCCAATTTTTG
CDK12 Sh3	CCGGATTTCCTGCATCGGGATATTACTCGAGTAATATCCCGATGCAGGAAATTTTTTG
CDK12 Sh4	CCGGCTATTGGTAAATCGCATTAAACTCGAGTTTAATGCGATTTACCAATAGTTTTTG

List of antibodies used in this study: All primary antibody incubations were overnight

RNA Pol II	SC-900
RNA Pol II pSer2	Bethyl A300-654A
PARP	CST 9532
RAD51	CST 8875
Cleaved Caspase 9	CST 9661
γ-H2AX	Millipore 05-636
H3	CST 4499
Alpha Tubulin	SC-8035
β actin	Sigma A5441
GAPDH	CST 2118
